# Expression of Mitochondrial-Encoded Genes in Blood Differentiate Acute Renal Allograft Rejection

**DOI:** 10.3389/fmed.2017.00185

**Published:** 2017-11-01

**Authors:** Silke Roedder, Tara Sigdel, Szu-Chuan Hsieh, Jennifer Cheeseman, Diana Metes, Camila Macedo, Elaine F. Reed, H. A. Gritsch, Adriana Zeevi, Ron Shapiro, Allan D. Kirk, Minnie M. Sarwal

**Affiliations:** ^1^Department of Clinical Affairs, Transplantation Research, Immucor Inc., Norcross, GA, United States; ^2^Department of Surgery, University of California, San Francisco, San Francisco, CA, United States; ^3^Department of Surgery, Duke University School of Medicine, Durham, NC, United States; ^4^Thomas E. Starzl Transplantation Institute, University of Pittsburgh Medical Center, Pittsburgh, PA, United States; ^5^Immunogenetics Center, University of California, Los Angeles, Los Angeles, CA, United States

**Keywords:** kidney transplantation, acute rejection, gene expression analysis, mitochondria, biomarkers

## Abstract

Despite potent immunosuppression, clinical and biopsy confirmed acute renal allograft rejection (AR) still occurs in 10–15% of recipients, ~30% of patients demonstrate subclinical rejection on biopsy, and ~50% of them can show molecular inflammation, all which increase the risk of chronic dysfunction and worsened allograft outcomes. Mitochondria represent intracellular endogenous triggers of inflammation, which can regulate immune cell differentiation, and expansion and cause antigen-independent graft injury, potentially enhancing the development of acute rejection. In the present study, we investigated the role of mitochondrial DNA encoded gene expression in biopsy matched peripheral blood (PB) samples from kidney transplant recipients. Quantitative PCR was performed in 155 PB samples from 115 unique pediatric (<21 years) and adult (>21 years) renal allograft recipients at the point of AR (*n* = 61) and absence of rejection (*n* = 94) for the expression of 11 mitochondrial DNA encoded genes. We observed increased expression of all genes in adult recipients compared to pediatric recipients; separate analyses in both cohorts demonstrated increased expression during rejection, which also differentiated borderline rejection and showed an increasing pattern in serially collected samples (0–3 months prior to and post rejection). Our results provide new insights on the role of mitochondria during rejection and potentially indicate mitochondria as targets for novel immunosuppression.

## Introduction

Kidney transplantation is the treatment of choice for most patients with end-stage renal disease. While significant improvements in immunological matching, surgical techniques, and particularly in the development of potent immunosuppressive drugs have allowed for increased graft survival rates, acute renal allograft rejection (AR) still occurs in 10–15% of patients, presenting a major risk factor for chronic allograft dysfunction and graft loss.

Kidney AR is a very complex process consisting of both cellular (lymphocyte mediated) and humoral (antibody mediated) mechanisms. While antigen-dependent adaptive immune mechanisms are critical to induce both AR and chronic AR, it has been recognized that antigen-independent injury and subsequent inflammation can precipitate and further enhance graft rejection, potentially orchestrated *via* modulation of the adaptive immune response by the recipient’s innate immune system. Further understanding of these rejection processes will allow for the development of more specific immunosuppressive drugs and more sensitive and specific diagnostics.

Donor brain death, ischemia during organ harvest, and subsequent graft reperfusion in the recipient can cause antigen-independent graft injury and inflammation and represent known risk factors for AR and chronic rejection (1, 2). In addition, endogenous triggers, also known as damage-associated molecular patterns (DAMPS) exist both intra- and extracellular, and can initiate and perpetuate a noninfectious, inflammatory response leading to graft injury, modulate the adaptive immune system, and enhance the development of AR (3). For example, extracellular haptoglobin has been shown to promote priming of allogeneic T-cells and experimentally enhanced rejection (4, 5). Similarly, mitochondrial components represent intracellular triggers of innate immune system-mediated inflammation (sterile inflammation) and there is an emerging role of DAMPS derived from mitochondria during inflammation (6, 7). Though they have not yet been causally linked to AR (3), they are critically involved in the regulation of T cell activation, expansion (8), and the development of memory T cells (9). Furthermore, cellular changes in mitochondria underlie ischemia reperfusion injury (IRI) (2), and mitochondria-induced cardiomyocyte death was observed during experimental cardiac AR providing a link to immune cell graft infiltration (10). Thought to originate from bacteria, mitochondria possess their own circular chromosome containing DNA encoding for 13 genes for subunits of the respiratory complexes I, III, IV, and V, and for 22 mitochondrial transfer RNA as well as for 2 ribosomal RNA (rRNA) (11). Under physiological conditions, mitochondria generate most of a cell’s ATP supply, are involved in cell signaling, cellular differentiation, and cell death, and playing a central role in inducing apoptosis (12). While the expression of nuclear encoded mitochondrial genes in renal allograft biopsies has been linked to allograft dysfunction and poor allograft outcome (13), the peripheral expression of the mitochondrial genome13 protein encoding genes in transplantation and during rejection has yet to be defined. In the present exploratory study, we investigated mitochondrial DNA encoded gene expression in kidney transplantation and in AR for the first time. Using quantitative real-time PCR (qPCR), we analyzed 155 peripheral blood (PB) samples collected from 115 unique renal transplant recipients from 4 transplant centers in the U.S. for the expression of 11 mitochondrial DNA encoded genes, 9 encoded on the respiratory complexes I, III, IV, and V, and 2 rRNA, of which the mt-7s represented the origin of replication containing several promotor sites for mRNA transcription.

## Materials and Methods

### Patients and Samples

Patients included in this study were enrolled from transplant programs at 4 different sites in the U.S.: University of California Los Angeles (UCLA), Emory University (Emory), University of Pittsburgh Medical Center (UPMC), and Stanford University (Stanford). All patients gave written informed consent, and the study was approved by the individual institutional review boards, and all procedures were conducted according to the principles expressed in the Declaration of Helsinki (14). Patients from Stanford were pediatric (<21 years) and patients from UCLA, Emory, and UPMC were adult (>21 years). Patient PB samples were paired with a contemporaneous renal allograft biopsy (bx) from the same patient within 48 h. Protocol bx were performed on all Stanford patients at engraftment, 3, 6, 12, and 24 months posttransplantation and additionally at the times of clinically suspected graft dysfunction (15); bx from patients at UCLA, Emory, and UPMC were performed per clinical indication at the point of suspected graft dysfunction based on a rise in serum creatinine >20% baseline. A conclusive phenotypic diagnosis was provided for same patient multiple PB–biopsy pairs utilized in this study. Multiple PB-biopsy pairs from the same patient were available from patients at Emory and UCLA. Sequential samples that were collected from the same patient during visits before and/or after a visit for a rejection biopsy were available from some patients at UCLA. Each biopsy was scored by the site’s pathologist using the latest Banff classification criteria for renal allograft pathology (16). PB samples collected at the point of clinically indicated bx were included in the analyses if obtained prior to any immunosuppression treatment intensification.

### Blood Sample Collection and RNA Extraction

Blood was collected in 2.5 ml PAXgene™ Blood RNA Tubes (PreAnalytiX, Qiagen, Valencia, CA, USA), in heparin or sodium citrate anticoagulant coated tubes (BD Vacutainer^®^ CPT™ Mononuclear Cell Preparation Tube—Sodium Citrate, BD Biosciences, San Jose, CA, USA) for peripheral blood mononuclear cell (PBMC) isolation. Total RNA was extracted using the column based method kits of PreAnalytix (Qiagen) for PAXgeneTM tubes and RNeasy (Qiagen) for PBMC isolation from Heparin coated tubes as per manufactures protocol. On-column DNAse treatment was performed to eliminate genomic DNA contamination in all samples. PBMC from sodium citrate coated CPT tubes were separated as described in the product insert for BD Vacutainer CPT, washed, and counted. PBMCs were subsequently resuspended in 90% fetal bovine serum (FBS; Gemini, Sacramento, CA, USA) and 10% dimethylsulfoxide (DMSO; Sigma, St. Louis, MO, USA) solution, and progressively cooled overnight to −80°C and subsequently transferred to liquid nitrogen or −150°C for storage. Quantity of total RNA was measured by NanoDrop spectrophotometer (NanoDrop).

### Complementary DNA (cDNA) Synthesis and Microfluidic qPCR Preparation

Complementary DNA synthesis was performed using 250 ng of extracted quality mRNA from the PB samples using the SuperScript^®^ II first strand cDNA synthesis kit (Invitrogen, Carlsbad, CA, USA) as per the manufacturers protocol. Samples were prepared for microfluidic qPCR analysis of 11 mitochondrial DNA encoded genes plus 18S by taking 1.52 ng total RNA from cDNA synthesis and processing through specific target amplification and sample dilution as described by Fluidigm (Fluidigm, South San Francisco, CA, USA) using pooled individual ABI Taqman assays for each gene, excluding ribosomal 18S RNA. Briefly, specific target amplification was performed using 1.52 ng of cDNA with the pooled Taqman assays in multiplex with Taqman PreAmp Master mix (ABI) to 5 μl final volume, for 18 cycles in a thermal cycler (Eppendorf Vapo-Protect, Hamburg, Germany), then diluted 1:5 with sterile water (Gibco, Invitrogen, Carlsbad, CA, USA). Utilization of TaqMan chemistry provided amplification of specific qPCR products only.

### Mitochondrial Genome Encoded Genes Studied

**Figure 1:**
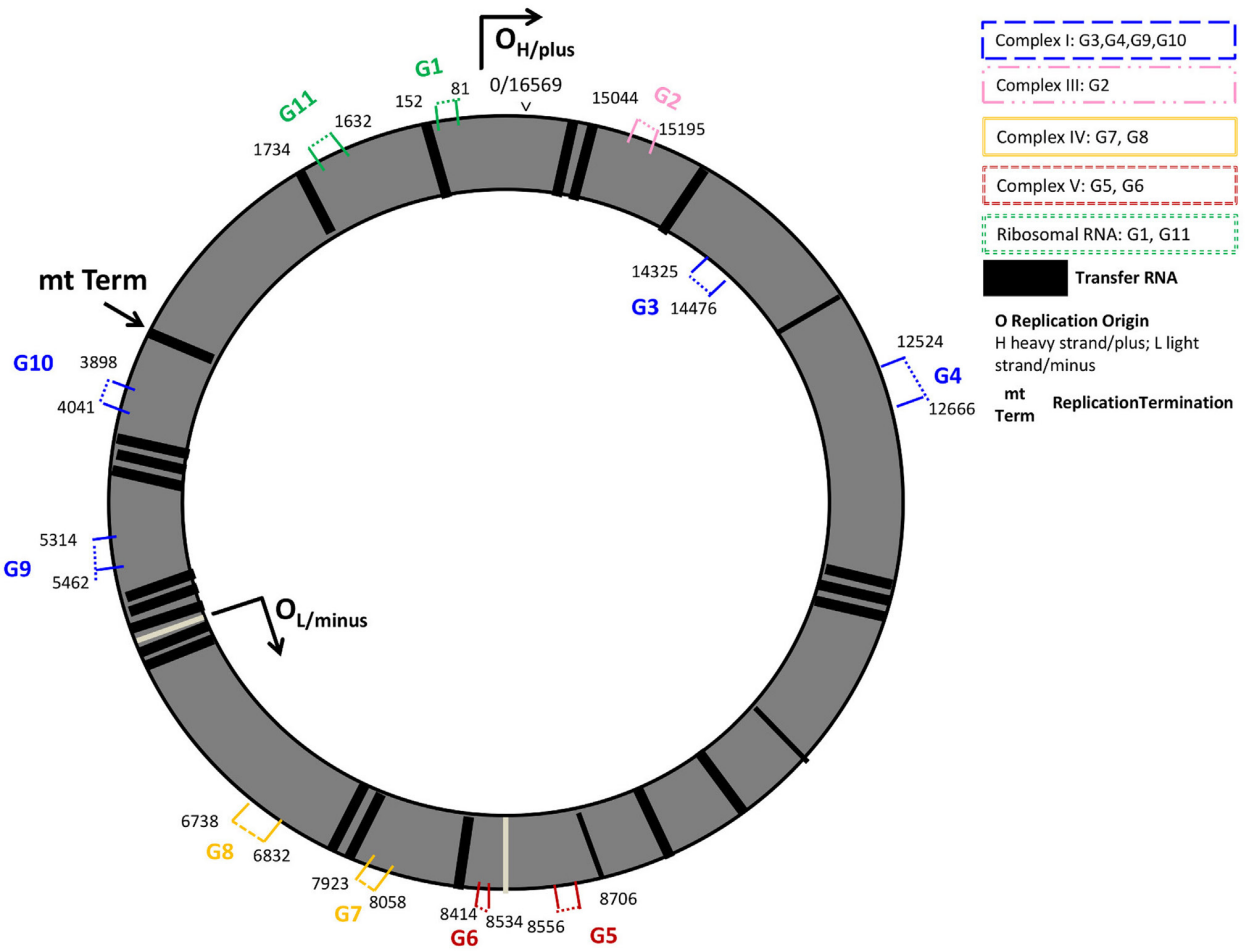
Mitochondrial genome regions studied in AR. Mitochondrial genes studied are numbered G1–G11 and colored by their location on structural genome complexes; numbers indicate the start and end base pairs: mt-7S/mt-D-loop (G1), and mt-12S (G11) represent ribosomal RNAs; mt-CYB (G2) the cytochrome b gene is located in the ubiquinol-cytochrome c reductase complex III; G3 (mt-ND6), G4 (mt-ND5), G9 (mt-ND2), G10 (mt-ND1) are sodium-ubiquinone oxidoredcutase chains (NADH; sodium dehydrogenase) located on Complex I (blue); mt-ATP6 (G5), and mt-ATP8 (G6) are adenosine-tri-phosphatases located on Complex V; cyclooxygenases mt-COII (G7) and mt-COI (G8) are located on Complex IV. G1, G2, G4–G11 are located on the outer, heavy DNA strand (O_H_), G3 is located on the inner, light DNA strand (O_L_). Black vertical lines represent transfer RNAs.

In the present study, we investigated the expression of 9 protein coding- and 2 rRNA-mitochondrial (mt-) DNA encoded genes. Protein coding genes were part of the mt-genome structural subunits complexes I, III, IV, and V. Complex I genes included the sodium-dehydrogenases NADH-6, -5, -2, and -1 (G3, G4, G9, G10); cytochrome B (G2) was studied from Complex III, the cyclooxygenases COX-1 and COX-2 (G8, G7) were studied from complex IV, and complex V encoded genes included in the analyses were the adenosine-tri-phosphatases ATPase-6 (G5), and -8 (G6). Additionally, we investigated the expression of the two rRNAs present on the mitochondrial genome rRNA-12s (G11), and the rRNA-7s (G1), known as the mt D-Loop, which is the origin of the mt-DNA replication and includes several promotor regions for mt-DNA transcription. Except for G3, which was encoded on the inner mt-DNA strand (L-strand), all genes studied were encoded on the outer mt-DNA strand (H-strand, G1, G2, and G4-G11) (Figure 1; Table S1 in Supplementary Material).

### Microfluidic qPCR

Microfluidic qPCR was performed on the Fluidigm BioMark (Fluidigm Inc., South San Francisco, CA, USA) instrument. Two dynamic qPCR microfluidic chip formats were utilized: 96.96 and 48.48, representing 96 gene × 96 sample and 48 gene × 48 sample, respectively. For qPCR, 2.25 µl of the diluted cDNA sample from the specific target amplification, along with Taqman Assays for each mitochondrial gene, Taqman Universal master mix and Fluidigm Loading reagent as outlined in the manufactures protocol were used, by priming and loading the microfluidic chips *via* the HX IFC Controller (Fluidigm) and performing the qPCR in the BioMark (Fluidigm), with default parameters for gene expression data collection, as advised by Fluidigm, including a linear derivative baseline correction to account for unequal efficiencies of target and control genes.

### Data Normalization and Pre Processing

Raw Ct data normalized using the delta delta Ct method against 18S and a human universal reference RNA was uploaded into Partek Genomics Suite v.6.6 (Partek Inc., St. Louis, MO, USA). Analysis of covariance and unsupervised principal component analysis were done for confounder analysis with a *p*-value <0.05 considered a significant association. RNA source (PBMC, PaxGene) was embedded in Transplant center as Stanford University collected PaxGene tubes only, and Emory, UPMC, and UCLA collected PBMC. Data were corrected for confounding variables by a multiple-way ANOVA. Included factors were experiment date, QPCR microfluidic chip format and transplant center, which embedded differences in sample collection methodology, donor and recipient age, donor type, and time posttransplant.

### Statistical Analysis

All data are presented as mean ± SEM or SD. Groups were compared using χ^2^-test for categorical variables, one- or multiple-way ANOVA with Tukey’s test to correct for multiple comparisons, two-sided Student’s *t*-test with Welch’s correction in case of unequal variances, and the nonparametric Kruskal–Wallis or Mann–Whitney *U*-test for non-normally distributed variables. The mitochondrial score (Mito-score) was defined as the geometric mean of expression of the mitochondrial DNA encoded genes in each sample. The statistical significance levels was defined as two-tailed *p*-value <0.05. All statistical analyses were performed in Partek Genomics Suite v.6.6., GraphPadPrim v.6.03 (GraphPad Software Inc.) and in Microsoft Excel (Microsoft, USA).

## Results

### Patients and Samples

A total of 155 PB samples were collected from 115 unique patients at Stanford (36/36 samples/patients), UCLA (62/24 samples/patients), Emory (28/26 samples/patients), and UPMC (29/29 samples/patients) and analyzed for mitochondrial gene expression (Table 1, Patient Demographics). Among the samples, 62 had matching biopsies that met Banff classification criteria for acute renal AR (16): 12 were classified as borderline rejection (BL-AR); 5 were antibody mediated (AMR) rejection with lesions in the glomeruli and peritubular capillary (PTC) compartments paired with positive C4d staining at PTC; 40 rejections were T-cell mediated (TCMR), and 5 showed a mixed type of AMR + TCMR rejection. Of the remaining samples, 60 samples were paired with a biopsy that did not reveal signs of significant abnormalities (No-AR); 10 samples were collected from patients that either showed acute tubular nephritis (*n* = 5), BK virus infection (*n* = 2), drug toxicity (*n* = 2), or thrombotic microangiopathy (*n* = 1). The remaining samples were serially collected from patients in the AR group, either up to 3 months prior to the rejection on biopsy (pre-AR, *n* = 13) and/or up to 3 months post the rejection on biopsy (post-AR, *n* = 10). Among all biopsies, 13 showed additional signs of chronic injury, defined as the presence of interstitial fibrosis/tubular atrophy (IF/TA) scores I–III. Mean creatinine levels in the AR group was 2.35 ± 1.5, and 1.29 ± 0.4 in the No-AR group (*p* < 0.0001). Mean glomerular filtration rates (GFR, Cockcroft–Gault estimation) were 38.23 ± 15.7 and 56.4 ± 111.2 in the AR and No-AR groups (*p* < 0.0001), respectively.

**Table 1:** Patient demographics included in the Study.

Parameters^a^	Stanford	Emory	University of California Los Angeles	University of Pittsburgh Medical Center	Total	*p*-Value
Samples/patients						0.0108
No. samples	36	28	62	29	155	
No. unique patients	36	26	24	29	115	
No. Contemp. Bx						
% AR/BL-AR	44.44	44.00	67.65	44.44		0.1484
# AR ≥ 1	9	11	23	7	*50*	
# BL-AR	7	0	0	5	*12*	
# No-AR	20	14	11	15	*60*	
Transplant type (% deceased)	26.67	37.50	40.32	87.50		0.0001
No. deceased	4	9	25	21	59	
No. living	11	15	37	3	66	
Time post-Txp (months)	11.15	59.33	6.93	31.00		<0.0001
SD	6.8	84.1	11.3	18.1		
Human leukocyte antigen (HLA) MM (x/6)	N/A	N/A	3.94	2.76		0.0025
SD	N/A	N/A	1.5	2.0		
Recipient gender (% male)	66.67	59.26	80.65	62.07		0.1175
No. male	20	16	50	18	104	
No. female	10	11	12	11	44	
Recipient age (years)	13.27	47.81	45.63	49.42		<0.0001
SD	5.4	16.2	9.5	18.2		
Donor gender (% male)	54.84	57.14	35.48	48.28		0.1841
No. male	17	12	22	14	65	
No. female	14	9	40	15	78	
Donor age (years)	27.06	37.27	40.89	46.96		<0.0001
SD	8.9	11.7	10.7	14.3		
Induction						<0.0001
# Thymo	0	28	62	0	90	
# Dac	36	0	0	0	36	
# Anti-CD52	0	0	0	29	29	
Primary IS	CNI, MMF, ±CS	CNI, MMF, CS	CNI, MMF, CS	CNI, MMF		
Sample collection	PAXgene	PBMC	PBMC	PBMC		
Centralized SOP	Yes	No	No	No		
Centrailzed RNA extraction	Yes	No	Yes	No		

*AR, acute rejection; BL, borderline; HLA MM, HLA mismatch; Contemp. Bx, contemporary renal allograft biopsy; Dac, Daclizumab; Thymo, thymoglobulin; CNI, calcineurin inhibitor; MMF, mycophenolate mofetil; CS, corticosteroids; IS, immunosuppression; PBMC, peripheral blood mononuclear cells*.

*^a^Numerical values represent mean plu/minus SD; p-Values for numerical values were calculated by 1-way ANOVA/Kruskal–Wallis test with Tukey’s/Dunn’s test for multiple comparisons, and by Student’s t-test; p-values for categorical values were calculated by Chi-Square test*.

### Renal Transplant Recipients >21 Years Have Significantly Higher Expression Levels of Mitochondrial Genome Encoded Genes

The primary focus of our study was to investigate the mitochondrial DNA encoded gene expression in AR. As mitochondria play a known role in aging, and our study included 36 samples from a pediatric (<21 years at time of transplantation, mean age = 13.27 ± 5.4) cohort, compared to 119 samples from an adult (>21 years at time of transplantation, mean age = 47.6 ± 14.6) in our study, we initially assessed the difference in mitochondrial gene expression between these cohorts and compared 36 pediatric samples to 119 adult samples and observed a marked increase of expression levels of all mt-genes (all *p*-values except for mt-G1 ≤ 0.05) in the adult cohort irrespectively of the pathology (Figure S1 in Supplementary Material). Due to the observed difference in mitochondrial DNA encoded gene expression with age, we focused our further analyses of mitochondrial gene expression during rejection in the adult and pediatric cohorts separately.

### Mitochondrial Encoded Genes Are More Abundantly Expressed in Adult and Pediatric Renal Transplant Patients Undergoing Rejection and They Distinguish Borderline from Rejection Grades I and Higher

**Figure 2:**
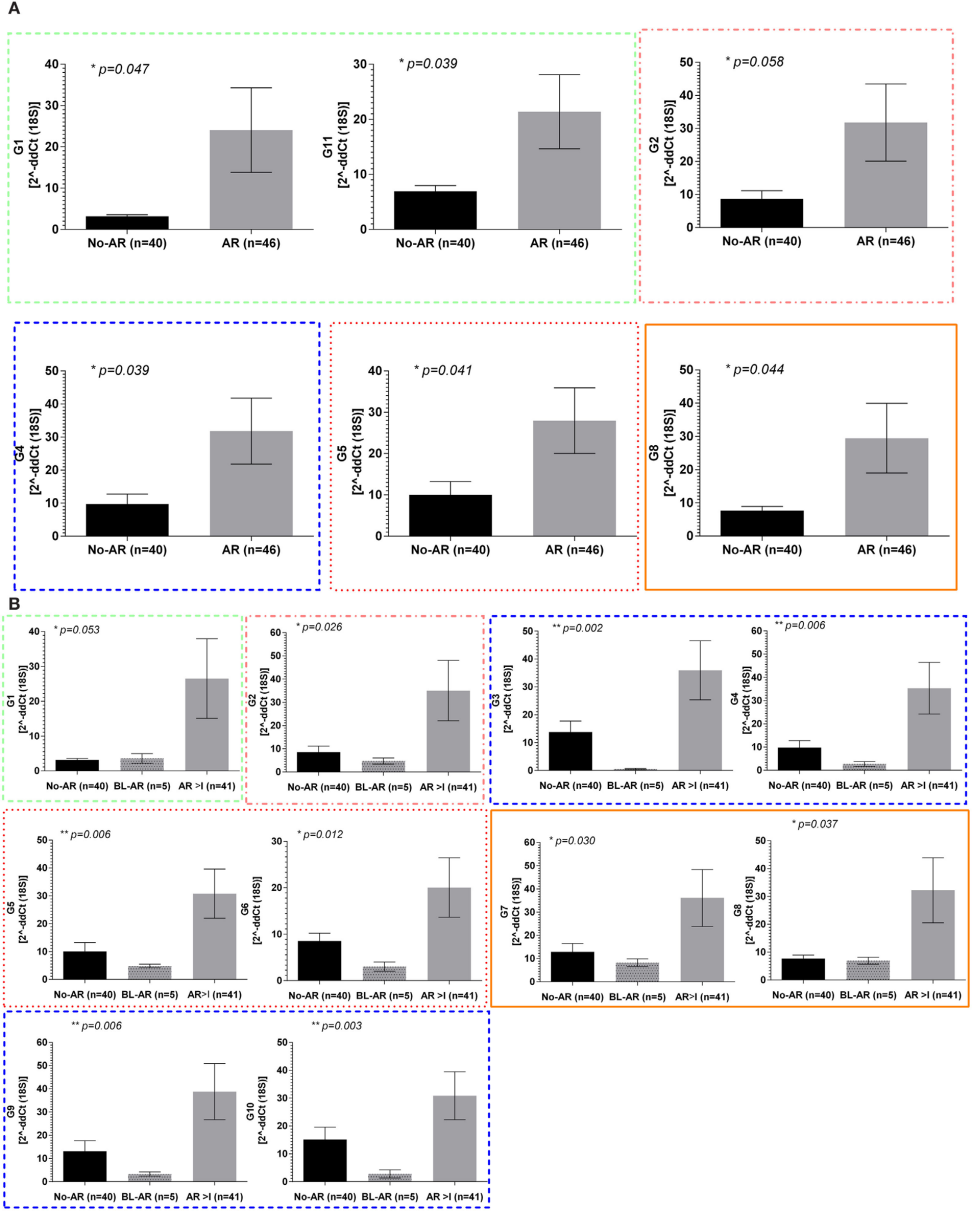
Mitochondrial gene expression during rejection in adult renal transplant recipients. Mitochondrial gene expression levels in peripheral blood samples from the adult cohort with matched biopsies without significant abnormalities (No-AR) were compared to samples with matched biopsies showing any signs of acute rejection (AR ≥ I, BL-AR) in the adult cohort; **(A)** expression levels of G1, G11, G2, G4, G5, and G8 were significantly increased in rejection (AR ≥ I plus BL-AR) compared to No-AR, **(B)** separating 5 BL-AR cases from AR ≥ I showed significantly higher expression in AR > I for 10 of the 11 genes studied (G1-G10, *p* ≤ 0.05). Mean plus SEM are shown for relative quantification of gene expression levels; *p*-Values were calculated using 2-sided Student’s *t*-test with Welch’s correction in case of unequal variance and *p* ≤ 0.05 were considered significant; frame colors indicate the structural complexes on the mitochondrial genome.

In the adult cohort of 119 samples, 46 had matching biopsies with either borderline rejection (BL-AR, *n* = 5) or with AR ≥ grade I (AR ≥ 1, *n* = 41) (AR), and 40 samples had matching biopsies which did not show signs of abnormalities (No-AR). In samples with AR, the expression of all 11 mitochondrial encoded genes was increased with mitochondrial genes G1, G2, G4, G5, G8, and G11 being significant (*p* ≤ 0.05, Figure 2A). Next, we evaluated whether the expression of the mitochondrial encoded genes was additionally able to distinguish BL-AR from AR ≥ grade I. In fact, 10 of the 11 genes were significantly higher expressed in AR ≥ I compared to the cases that did not fully meet classification criteria of rejection (BL-AR vs. AR ≥ I; *p* ≤ 0.05; Figure 2B).

**Figure 3:**
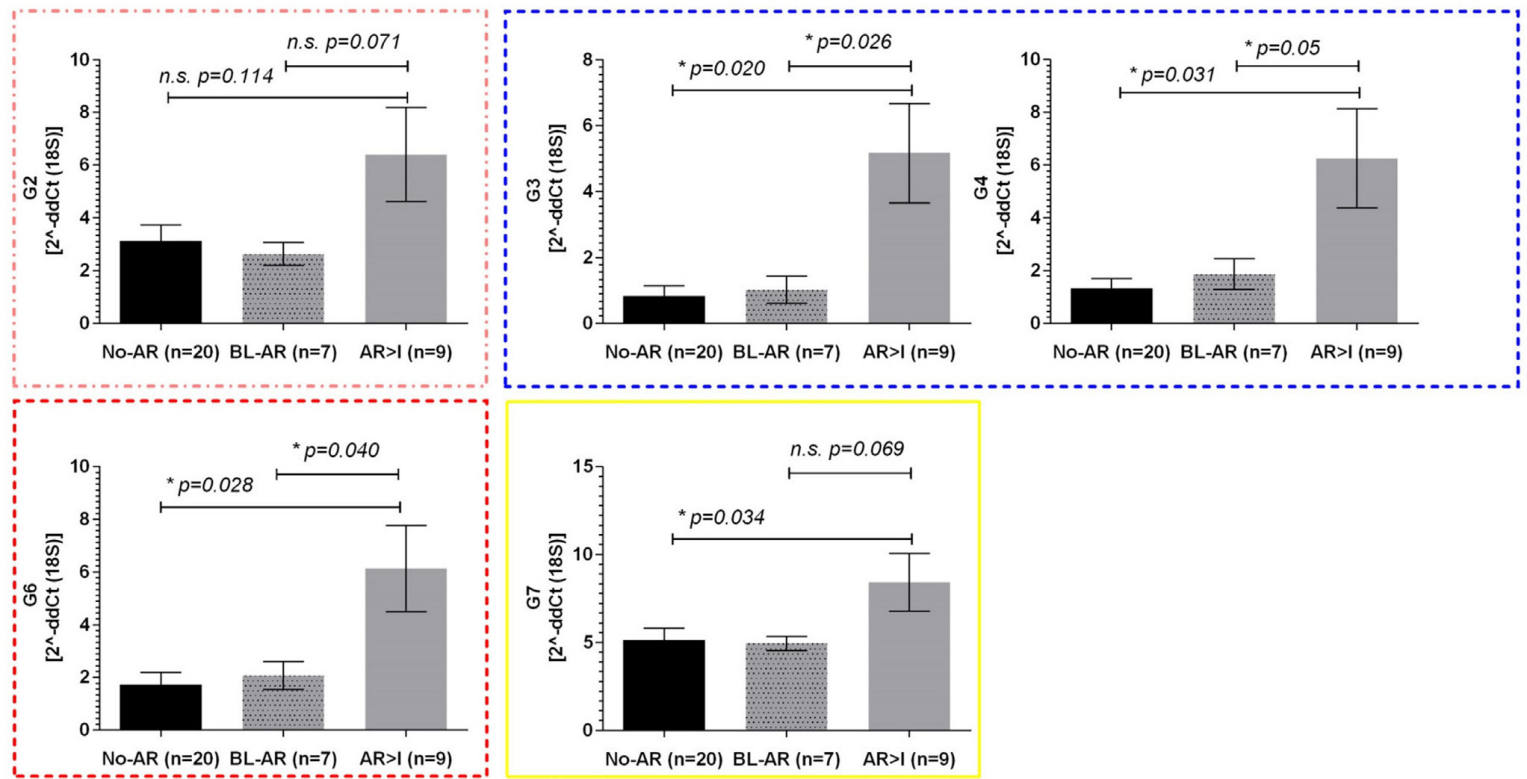
Mitochondrial gene expression in pediatric renal transplant recipients with BL-AR and AR ≥ I. Mitochondrial gene expression levels in peripheral blood samples from pediatric patients with matched biopsies without significant abnormalities (No-AR) were compared to samples with matched biopsies showing borderline rejection (BL-AR), and rejection grade I and higher (AR ≥ I); G2, G3, G4, G6, and G7 were significantly increased in AR ≥ I compared to No-AR and BL-AR. Mean plus SEM are shown for relative quantification of gene expression levels; *p*-values were calculated using 2-sided Student’s *t*-test with Welchs’s correction in case of unequal variances; *p* ≤ 0.05 were considered significant; frame colors indicate the structural complexes on the mitochondrial genome.

The pediatric cohort of 36 samples included 7 BL-AR, 9 AR ≥ I, and 20 No-AR. Similarly to the adult cohort, we observed elevated expression of mitochondrial genes during rejection in the pediatric cohort with significant differences between No-AR and AR ≥ I for genes G2, G3, G4, G6, and G7 (*p* ≤ 0.05). Again, these genes were also able to distinguish BL-AR from AR ≥ I (*p* ≤ 0.05) and showed similar expression between No-AR and BL-AR (*p* > 0.05) (Figure 3).

Whereas the pediatric cohort demonstrated significantly increased expression in rejection for genes G2, G3, G4, G6, and G7, the adult cohort showed significant upregulation for 10 out of 11 mitochondrial encoded genes studied when separating No-AR, BL-AR, and AR ≥ I (Figure 2B).

### Peripheral Mitochondrial Gene Expression Can Be Expressed As a Mito-Score

**Figure 4:**
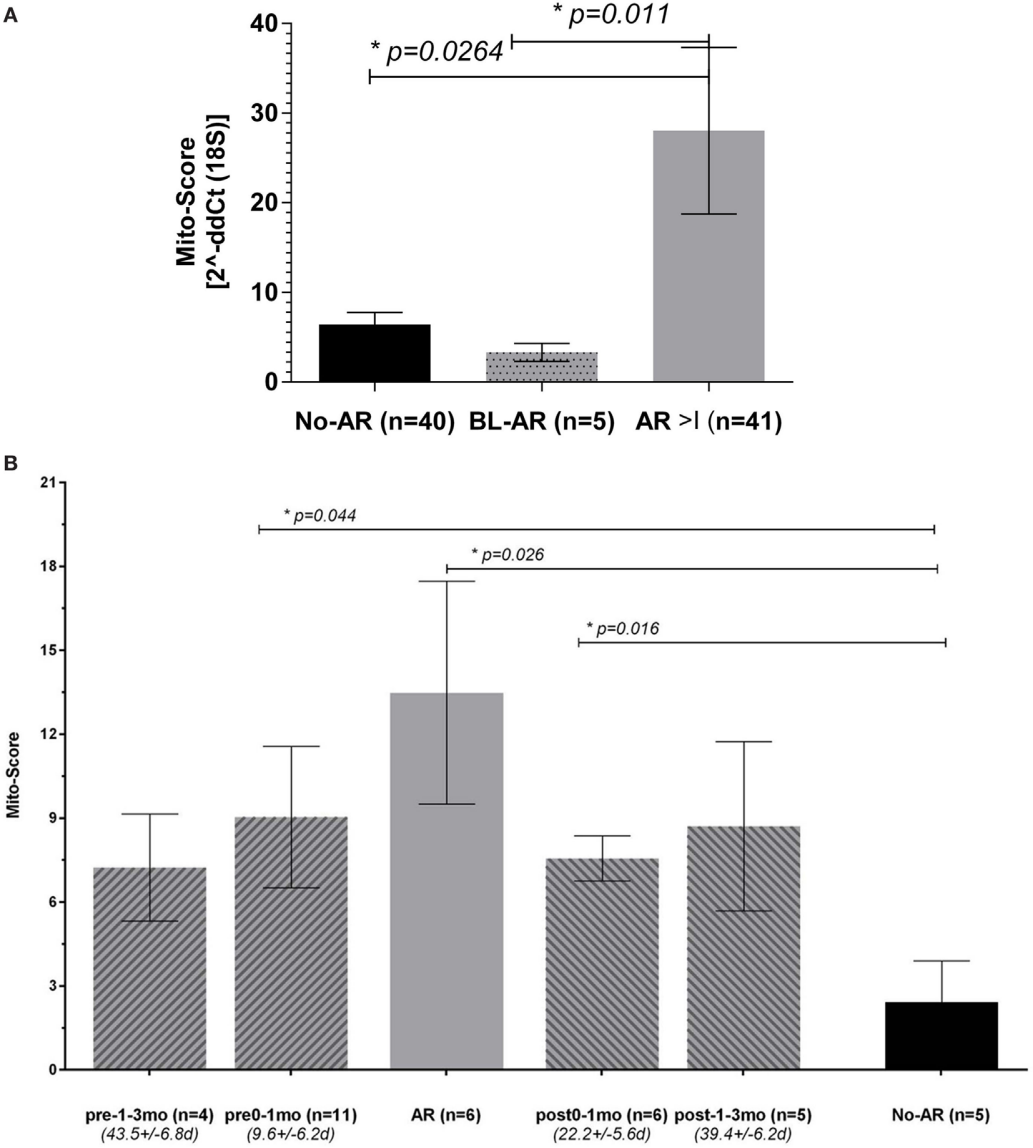
Peripheral mitochondrial gene expression can be summarized into a Mito-Score. **(A)** When summarized into a Mito-Score, the mitochondrial gene expression differentiated No-AR from BL-AR and AR ≥ I (No-AR vs. AR ≥ I, *p* = 0.026 and BL-AR vs. AR ≥ I, *p* = 0.011); **(B)** analysis of the Mito-Scores in serially collected samples revealed increased expression before and after a rejection. Serially collected samples were available from 17 adult patients. Samples were categorized according to their time of collection before and after an AR biopsyinto pre-1–3 months (*n* = 4, average time 43.5 ± 6.8 days), pre-0–1 months (*n* = 11, average time 9.6 ± 6.2 days), and into post-0–1 months (*n* = 6, average time 22.2 ± 5.6 days) and post 1–3 months (*n* = 5, average time 39.4 ± 6.2 days). Mito-Scores were compared to scores in matching samples at the point of No-AR (*n* = 5) by biopsy. Mito-Scores were significantly elevated in samples collected up to 1 month before and after the rejection when compared to the No-AR time-point. Mito-Scores are displayed as mean plus SEM; *p*-values were calculated using 2-sided Student’s *t*-test with Welch’s correction in case of unequal variances and *p* ≤ 0.05 was considered significant.

The observed upregulation of all mitochondrial DNA encoded genes in patients with AR led to the definition of a Mito-Score to evaluate the combined effect of these genes as the geometric mean of the RQ of the 11 mt-genes. We observed a significantly upregulated Mito-score in patients with AR compared to No-AR (*p* = 0.03); the Mito-score also allowed for distinction of BL-AR (*p* = 0.01) (Figure 4A). We also observed a slight positive correlation of the Mito-score at the point of biopsy with serum creatinine and a slight negative correlation of the Mito-Score with eGFR (milliliter per minute/.); however, these correlations did not reach significance (*p* > 0.05, Spearman Rank Correlation analysis).

### Analysis of the Dynamics in Mitochondrial Gene Expression Reveals Increased Expression before and after Biopsy Confirmed Acute Rejection

To evaluate the dynamics of the mitochondrial gene expression in rejection, we analyzed serially collected samples before and after an AR episode per biopsy. Serially collected samples were available from 17 patients from one center with samples either collected up to 3 months prior to and/or post the AR biopsy, or at times of No-AR. Available for the pre- 1–3 month group (mean time to rejection = 43.5 ± 6.8days) were 4 samples, available for the pre 0–1 month group (mean time to rejection = 9.6 ± 6.2days) were 9 samples; for the post 0–1 month (mean time from rejection = 22.2 ± 5.6 days), and post 1–3 month (mean time from rejection = 39.4 ± 6.2 days) groups, each five samples were available. Matching samples collected at the point of an AR episode by biopsy were available for 9 patients with 5 patients contributing 2 rejection samples, and matching samples with a biopsy of No-AR were available for 5 patients (note: not all 17 patients had a pre-, a post-AR, and a matching No-AR sample). While mito-scores were highest at the point of AR compared to all other groups, we observed an increase mito-score 0–1 month before and after the AR episode compared to the Normal samples (Figure 4B). Despite the marked increase observed, differences did not reach significance, probably due to small number of available samples in each group (*n* = 4, *n* = 5, respectively). The Mito-Score was not correlated with time post transplantation (*r* = 0.03, *p* = 0.859, Pearson Correlation).

### HLA Mismatched Patients Demonstrate Higher Levels of Mitochondrial Expression Which Are Further Increased during Rejection

We were interested whether the number of HLA mismatches (MM, A, B, DR) between donor and recipient would be associated with the level of mitochondrial gene expression. Information on number of HLA MM was available for 91 samples. We observed increased mitochondrial gene expression expressed as the mito-score in patients with >2 MM in HLA and with highest levels in patients with 4 HLA MM (Figure S2A in Supplementary Material). As in this group, the number of samples with AR was highest, we next differentiated the samples into 27 samples AR ≥ grade I, and 24 samples No-AR and created two HLA groups, group 1 included samples from patients with 0–3 HLA MM, and group 2 included samples from patients with 4–6 HLA MM. Mito-Scores were increased in AR and No-AR between the two groups and further increased in AR. Mean Mito-Scores in group 1 were 8.6 ± 7.2 for AR and 4.4 ± 5.4 for No-AR, mean Mito-Scores in group 2 were 15.9 ± 23.0 and 7.7 ± 7.1 for group AR and No-AR, respectively. Immunosuppression was similar in these patients consisting of a T-cell depletion regimen for induction and a calcineurin inhibitor, mycophenolate mofetil, with or without steroids for maintenance. Due to the low number of samples, levels did not reach the significance level of *p* < 0.05 (Student’s *t*-test with Welch correction) (Figure S2B in Supplementary Material).

## Discussion

Mitochondria represent unique cellular organelles most likely originating from bacteria via endosymbiosis and are often referred to as DAMPS, which are critically involved in inflammatory processes. These inflammatory processes, such as IRI, or regulations of T-cell activation play significant roles in the development of early transplant injury and of acute AR. The majority of findings about the role of mitochondria in inflammation resulted from cell-lines or experimental models, and only few studies have been actually performed in human samples. Mitochondria are unique in that they carry their own genome and gene expression machinery. The expression of these mitochondrial genome encoded genes in kidney transplantation and acute renal AR has still to be defined and could provide relevant insights into the rejection mechanisms to potentially provide for the development of novel immunosuppressive drugs and improved diagnostics. In the present study, we investigated PB samples for the expression of mitochondrial genome encoded genes in patients with and without acute rejection.

In a first step, we observed a difference of mitochondrial gene expression with recipient age with increased expression in adults (>21 years) compared to pediatrics (<21 years). The role of mitochondria in aging is well known and majorly seen in declining mitochondrial function and accumulation of mtDNA deletions (17–19). Increased levels of the mitochondrial Complex I and IV genes COX I, ND1, and ND5 were observed in platelets isolated from adult healthy subjects (69–82 years) when compared to levels in young healthy subjects (21–29 years) (20), supporting our findings. One could argue that this finding was subject to potential detection of DNA as a result of different blood collection methodologies. This potential was limited by performing DNAse treatment during the RNA extraction for all samples and by using TaqMan chemistry. Our subsequent separate analyses of adult and pediatric recipients demonstrated increased mitochondrial gene expression in acute rejection in both cohorts. Interestingly, when we separated the cases, which did not fully meet the Banff classification criteria for AR from the cases that fully met the rejection criteria, we observed lower expression levels in these borderline AR, which was more similar to the expression in the No-AR cases. The extent to which the additionally observed finding of lower BL expression compared to NoAR can be explained by the current study is limited as in contrast to the No-AR, and AR > I cases, the number of BL-AR cases was small (*n* = 5 in the adult and *n* = 7 in the pediatric cohort). In contrast to our analyses in serially collected samples, which were conducted in paired samples, these analyses were conducted in unpaired samples. An effect of individuality in gene expression patterns in human blood (including PAXgene and PBMC) was studied here (21), and the results revealed distinct patterns of interindividual variation, which could be traced to variation in the relative proportions of specific blood cell subsets in addition to gender, and age, additionally supporting our results. However, further analyses of the individual gene expression levels in the patients with a BL samples were limited as serial samples were not available from these patients. Our overall observed upregulation of mitochondrial gene expression in PB, however, may be linked to the immune-cell proliferation and production of effector molecules during rejection, which requires substantial alterations in cell metabolism, including generation of energy through mitochondria-dependent oxidative phosphorylation (22). Increased levels of mitochondrial gene expression have also been found in studies of colorectal cancer, where mt-ND6 and mt-ND1 mRNA levels were significantly increased in growing adenocarcinomas. The authors of this study suggested mitochondrial produced reactive oxygen species (mROS)-induced mutations in mtDNA as a cause for differential gene expression. mROS production has also been closely linked to the development of acute AR *via* innate immunity (23), as well as to prolonging antigen-specific proliferative responses in T cells (24). Thus, increased levels of mitochondrial genes may indicate the development of mROS-induced graft injury and potentially acute rejection, an assumption, which was supported by our findings in serially collected samples where we observed increasing expression levels in samples collected up to 3 months prior to the rejection. While the expression decreased post the AR episode, it remained elevated when compared to NoAR, which potentially indicates persistent immune activation in these patients. Additional follow-up samples, optimally matched with a NoAR biopsy were not available to support this finding of suggested unresolved immune activation. Zepeda-Orozco et al. (13) found an association between impaired mitochondria biogenesis pathways and gene expression in allograft biopsies with interstitial fibrosis and inflammation. Of note is that the authors of this study investigated allograft biopsies and applied microarray technology, which in contrast to our investigations assessed genomic DNA encoded mitochondrial gene expression as compared to mitochondrial genome encoded mitochondrial gene expression. Considering both findings, one could hypothesize that an impaired mitochondrial biogenesis resulting in fewer numbers of mitochondria, may actually result in increased activity of remaining mitochondria. However, whether impaired mitochondrial biogenesis in tissue can be related to an increased mitochondrial gene expression in blood at points of inflammation cannot be assumed given the present findings.

Whether the observed rather non selective upregulation of all mitochondrial genes during rejection in this study can be further specified to selective mitochondrial genes and their function are included in additional analyses, which are currently under way. The profiling of patients with different immune effector mechanisms of rejection will further elucidate differences in mitochondrial gene expression as well as whether mitochondrial proteins may represent non-HLA antigens. We admit that the number of serially collected samples in our study was limited and additional samples will be needed to further investigate the long-term outcome of patients showing persistent upregulation of mitochondrial gene expression.

In summary, we observed increased expression of mitochondrial genes in PB from patients with acute renal AR, compared to patients without rejection. Our results provide additional insights into the rejection processes and suggest that mitochondrial gene expression responses of circulating leukocytes can potentially provide an early warning of inflammatory processes during rejection, which may be mediated by other than the classical adaptive immune mechanisms. The development of drugs targeting mitochondrial mechanisms including mROS production might inhibit the development of rejection escaping current immunosuppression.

## Ethics Statement

Patients included in this study were enrolled from transplant programs at four different sites in the U.S.: University of California Los Angeles (UCLA), Emory University (Emory), University of Pittsburgh Medical Center (UPMC), and Stanford University (Stanford). All patients gave written informed consent. The study was approved by the individual institutional review boards, and all procedures were conducted according to the principles expressed in the Declaration of Helsinki.

## Author Contributions

Conceived and designed the experiments: MS, SR. Performed the experiments: SCH, SR. Analyzed the data: SR, MS. Contributed reagents/materials/analysis tools: DM, AZ, HG, JC, CM, RS, MS, AK, ER, MS, TS. Wrote the first draft of the manuscript: SR, MS. Wrote the paper: SR, MS.

## Conflict of Interest Statement

The authors declare that the research was conducted in the absence of any commercial or financial relationships that could be construed as a potential conflict of interest.

## Supplementary Material

Table S1 Details of mitochondrial genes studied.

Figure S1 Comparison of mitochondrial genome encoded gene expression in adult (>21 years) and pediatric (<21 years) kidney transplant recipients.

Figure S2 **(A)** Mito-score levels in renal transplant recipients with 0–6 human leukocyte antigen (HLA) mismatched antigens. **(B)** Mito-score levels between AR and No-AR categorized by the number of HLA mismatched antigens into 0–3 and 4–6 mismatches.

## Abbreviations

AR, acute rejection; AMR, antibody mediated rejection; ATN, acute tubular nephritis; ATP, adenosine-tri-phosphatase; Basi, basiliximab; BL, borderline acute rejection; CNI, calcineurin inhibitor; COX, cyclooxygenase; CS, corticosteroids; CYB, cytochrome B; DAC, daclizumab; DAMPS, damage-associated molecular patterns; GFR, glomerular filtration rate; HLA, human leukocyte antigen; IF/TA, interstitial fibrosis/tubular atrophy; IRI, ischemia reperfusion injury; IS, immunosuppression; MMF, mycophenolate mofetil; Mt, mitochondrial; NADH, sodium-dehydrogenase; No-AR, absence of rejection on biopsy; PB, peripheral blood; PBMC, peripheral blood mononuclear cells; PTC, peritubular capillary; Thymo, thymoglobuline.
