# Supplementary material for: Expression of Mitochondrial-Encoded Genes in Blood Differentiate Acute Renal Allograft Rejection
Source: Front Med (Lausanne). 2017 Nov 1;4:185. doi: 10.3389/fmed.2017.00185 (PMC5671971; doi:10.3389/fmed.2017.00185)

**Supporting Information**

**Table S1:** Details of mitochondrial genes studied

| **Assay ID** | **Gene Symbol** | **EntrezID** | **Gene Name** | **GenBank mRNA** |
| --- | --- | --- | --- | --- |
| Hs02596863_g1 | MT-ATP8;- | 4509 | mitochondrially encoded ATP synthase 8 | NC_012920.ATP8.0 |
| Hs02596873_s1 | MT-ND1;- | 4535 | mitochondrially encoded NADH dehydrogenase 1 | NC_012920.ND1.0 |
| Hs02596864_g1 | MT-CO1;- | 4512 | mitochondrially encoded cytochrome c oxidase I | NC_012920.CO1.0 |
| Hs02596874_g1 | MT-ND2;- | 4536 | mitochondrially encoded NADH dehydrogenase 2 | NC_012920.ND2.0 |
| Hs02596867_s1 | MT-CYB;- | 4519 | mitochondrially encoded cytochrome b | NC_012920.CYB.0 |
| Hs02596859_g1 | MT-RNR1;- | 4549 | mitochondrially encoded 12S RNA | NC_012920.RNR1.0 |
| Hs02596878_g1 | MT-ND5;- | 4540 | mitochondrially encoded NADH dehydrogenase 5 | NC_012920.ND5.0 |
| Hs02596879_g1 | MT-ND6;- | 4541 | mitochondrially encoded NADH dehydrogenase 6 | NC_012920.ND6.0 |
| Hs02596862_g1 | MT-ATP6;- | 4508 | mitochondrially encoded ATP synthase 6 | NC_012920.ATP6.0 |
| Hs02596865_g1 | MT-CO2;FTH1 | 4513 | mitochondrially encoded cytochrome c oxidase II;ferritin; heavy polypeptide 1 | AY258285.1;NC_012920.CO2.0 |
| Hs02596861_s1 | MT-7S;- | MT-D-Loop | AB Mitochondrial Gene 7S; encoding D-loop which is a replication start site of the mtDNA | NC_012920.7S.0 |

**Figure S1:** Comparison of mitochondrial genome encoded gene expression in adult (>21 years) and pediatric (<21 years) kidney transplant recipients

**
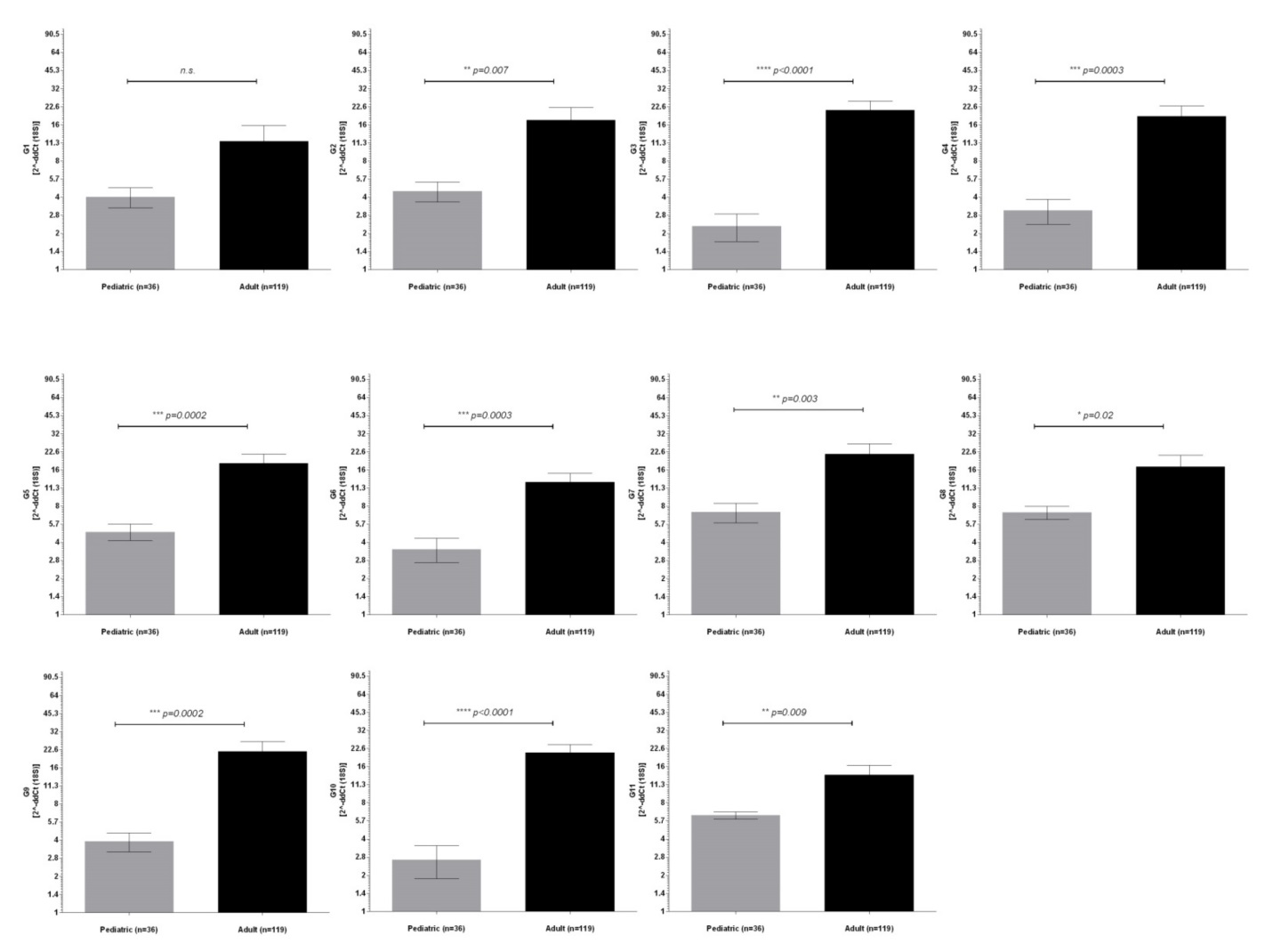
**

**Figure S2: S2a)** Mito-Score levels in renal transplant recipients with 0-6 HLA mismatched antigens;


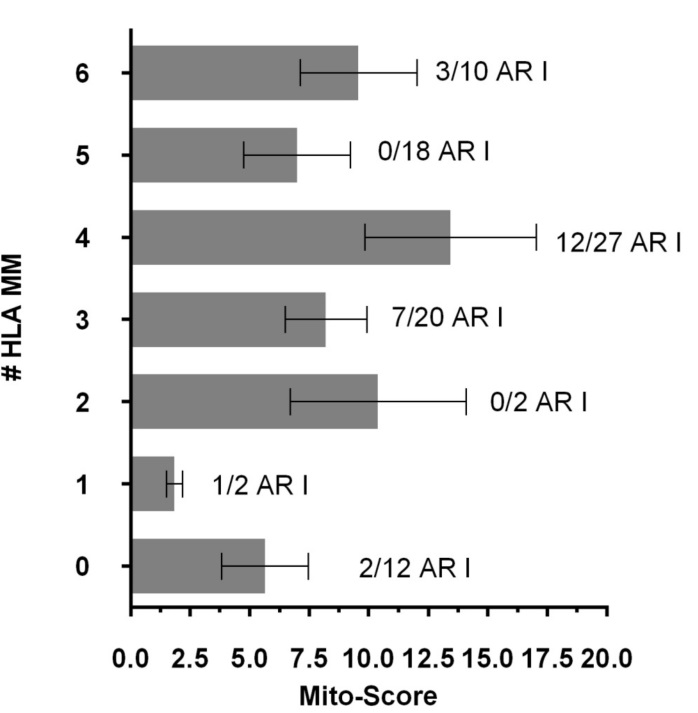


**S2b)** mito-score levels between AR and No-AR categorized by the number of HLA mismatched antigens into 0-3 and 4-6 mismatches. Numbers on top of the graphs represent numbers of patients in each category.


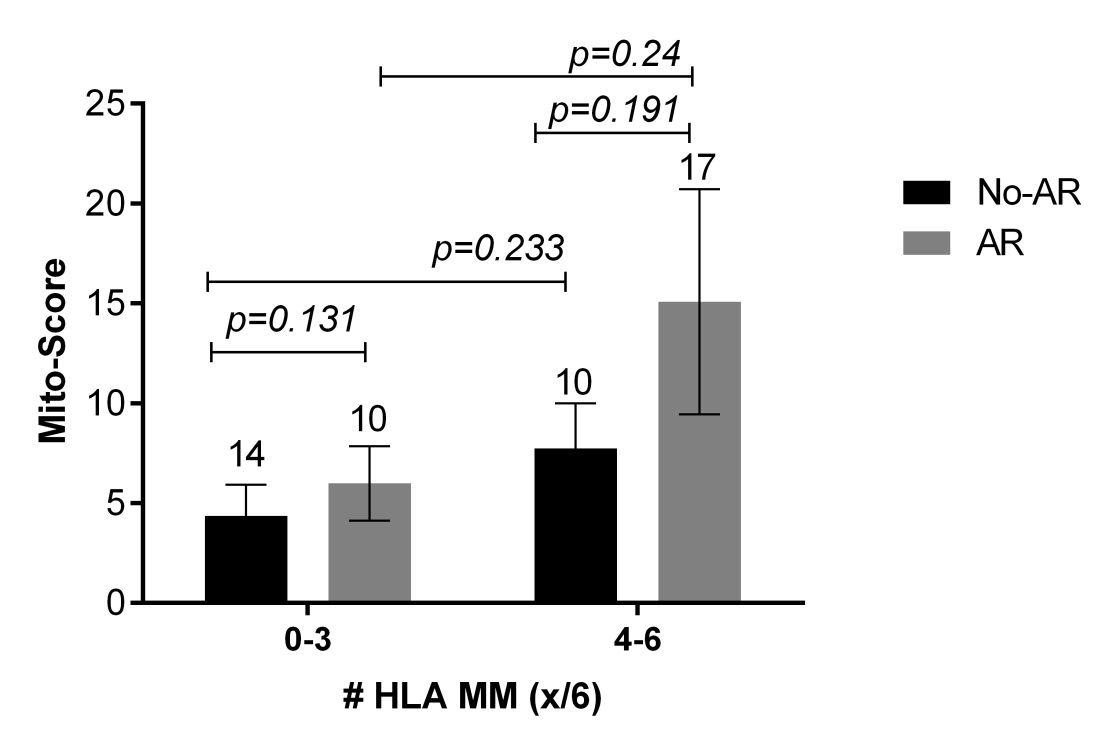

Supplement: Table S1 — Details of mitochondrial genes studied. [file data_sheet_1.docx]
